# Functional Food Targeting the Regulation of Obesity-Induced Inflammatory Responses and Pathologies

**DOI:** 10.1155/2010/367838

**Published:** 2010-05-25

**Authors:** Shizuka Hirai, Nobuyuki Takahashi, Tsuyoshi Goto, Shan Lin, Taku Uemura, Rina Yu, Teruo Kawada

**Affiliations:** ^1^Laboratory of Molecular Function of Food, Division of Food Science and Biotechnology, Graduate School of Agriculture, Kyoto University, Uji, Kyoto 611-0011, Japan; ^2^Department of Food Science and Nutrition, University of Ulsan, Ulsan 680-749, Republic of Korea

## Abstract

Obesity is associated with a low-grade systemic chronic inflammatory state, characterized by the abnormal production of pro- and anti-inflammatory adipocytokines. It has been found that immune cells such as macrophages can infiltrate adipose tissue and are responsible for the majority of inflammatory cytokine production. Obesity-induced inflammation is considered a potential mechanism linking obesity to its related pathologies, such as insulin resistance, cardiovascular diseases, type-2 diabetes, and some immune disorders. Therefore, targeting obesity-related inflammatory components may be a useful strategy to prevent or ameliorate the development of such obesity-related diseases. It has been shown that several food components can modulate inflammatory responses in adipose tissue via various mechanisms, some of which are dependent on peroxisome proliferator-activated receptor *γ* (PPAR*γ*), whereas others are independent on PPAR*γ*, by attenuating signals of nuclear factor-*κ*B (NF-*κ*B) and/or c-Jun amino-terminal kinase (JNK). In this review, we introduce the beneficial effects of anti-inflammatory phytochemicals that can help prevent obesity-induced inflammatory responses and pathologies.

## 1. Introduction

Recently, more and more lines of evidence have accumulated that obesity is associated with low-grade chronic inflammation that is causally involved in the development of insulin resistance. Systemic inflammation is markedly evident in a number of human and mouse models of obesity, as determined by increased plasma levels of inflammatory cytokines such as tumor necrosis factor-*α* (TNF-*α*), interleukin-6 (IL-6), and monocyte chemoattractant protein-1 (MCP-1). These inflammatory cytokines are derived from obese adipose tissue [1], and recently, it has been found that not only adipocytes, but also immune cells, such as macrophages [2, 3] reside in adipose tissue, and that these cells may induce insulin resistance by promoting inflammation in these tissues. The major cause of the development of obesity and the consequent inflammatory disorders is the excess dietary fat intake or an imbalance between the intake and expenditure of energy. Overweight and obese patients may develop paradoxical nutritional deficiency from eating high energy foods with poor nutrient content; however, diet with a higher nutrient density reduces their weight and improves obesity-related inflammatory disorders [4]. This indicates that obesity-related pathologies can be prevented or improved by the intake of food containing components that can control inflammation in obese adipose tissues infiltrated with macrophages. In activated macrophages, inflammatory responses are regulated by master regulators of inflammation such as nuclear factor-*κ*B (NF-*κ*B) and c-Jun amino-terminal kinase (JNK) [5, 6]. Moreover, peroxisome proliferator-activated receptor-*γ* (PPAR*γ*) is reported to attenuate inflammation in activated macrophages by interfering with NF-*κ*B signaling [7]. Therefore, targeting these inflammatory regulators using food components may be a useful strategy to prevent or ameliorate the development of obesity-related diseases. Our group and other research groups have shown that several food components can modulate inflammatory responses in adipose tissue via various mechanisms, some of which are dependent on PPAR*γ*, whereas others are PPAR*γ*-independent, by attenuating NF-*κ*B or JNK signaling. In this review, we introduce the beneficial effects of anti-inflammatory food components against obesity-induced inflammatory responses and pathologies.

## 2. Inflammatory Components Associated with Obesity and Related Pathologies

**Figure 1:**
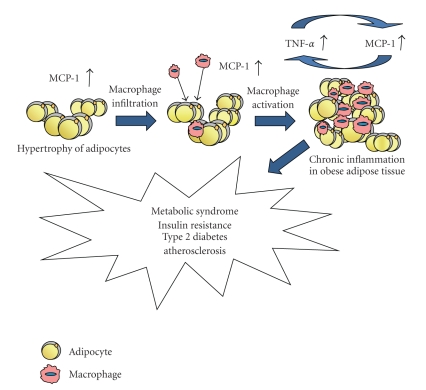
The development of vicious cycle of inflammation between adipocytes and macrophages in obese adipose tissue.

Adipose tissue is composed of adipocytes and stromal vascular cells containing various cell types such as preadipocytes, endothelial cells, fibroblasts, and numerous immune cells. In particular, macrophage infiltration into adipose tissue is prominent in obesity, and the number of macrophages in adipose tissue correlates with body mass index, adipose size, and the total amount of body fat [2, 3]. It has been suggested that adipose tissue-derived MCP-1, a CC chemokine that exhibits chemotactic properties on inflammatory cells, is the key factor for inducing macrophage infiltration into adipose tissue. The level of MCP-1 released by adipocytes is significantly greater in obese mice than in nonobese mice [8–10] and is markedly increased when adipocytes are cocultured with macrophages [11, 12]. MCP-1 from hypertrophic adipocytes in obese adipose tissue can also trigger macrophage infiltration into adipose tissue and subsequently activates macrophages to release inflammatory mediators such as TNF-*α* [10], which interferes with insulin signaling and induces fatty acid lipolysis in adipocytes. The concentrations of these fatty acids, particularly saturated free fatty acids, are reported to be elevated in obesity [13] and directly induce inflammatory responses in macrophages via toll-like receptor 4 (TLR4), the lipopolysaccharide receptor [14, 15]. The NF-*κ*B and JNK pathways represent important modulators of inflammatory gene expression downstream of TLR4 in many cell types, including macrophages [11, 16, 17]. In this way, adipocytes and macrophages interact in a paracrine manner and create a vicious cycle of inflammation that augments the inflammatory changes and insulin resistance in obese adipose tissue [11] (Figure 1).

## 3. Strategy to Prevent Inflammatory Responses and Insulin Resistance in Obese Adipose Tissues by Food Components

**Figure 2:**
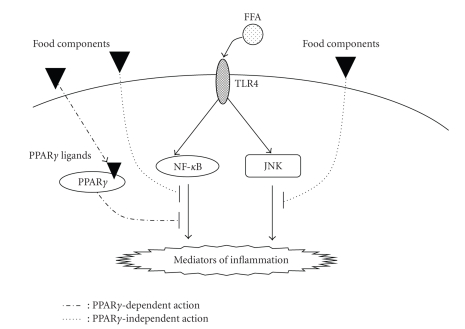
Signaling pathway of inflammatory gene expressions in obese adipose tissue and the strategy to prevent the obese-related pathologies by food components.

Inflammatory responses in obese adipose tissues are regulated by many transcriptional factors. NF-*κ*B and JNK represent important modulators of inflammatory gene expression downstream of TLR4 in adipose tissues, suggesting that food components interfering with the TLR4/NF-*κ*B or TLR4/JNK axis could be useful to prevent the onset of insulin resistance in obese patients (Figure 2).

Furthermore, PPAR*γ*, a member of the nuclear receptor superfamily activated by ligands, also plays an important role in inflammation [18, 19]. Thiazolidinediones (TZDs), synthetic ligands for PPAR*γ*, suppress the production of proinflammatory cytokines including TNF-*α* in LPS-stimulated macrophages [7]. In addition to the anti-inflammatory effect, TZDs regulate the mRNA expression of genes involved in lipid metabolism in macrophages and suppress their transformation into foam cells [7, 20]. On the other hand, TZDs have been widely used as antidiabetic drugs, which activate PPAR*γ* to resulting in the promotion of adipocyte differentiation [21]. TZDs not only stimulate glucose uptake into differentiated adipocytes but also induce the production of adiponectin, an insulin-sensitivity-promoting factor [22], and suppression of TNF-*α* through the PPAR*γ* activation in adipocytes [23]. Thus, food components that act as ligands for PPAR*γ* can show multiple effects, including anti-diabetes and anti-inflammatory effects. Currently, two different molecular mechanisms have been proposed by which the anti-inflammatory actions of PPAR*γ* are in effect: (1) via interference with proinflammatory transcription factors including NF-*κ*B [7], and (2) by preventing the removal of corepressor complexes from gene promoter regions resulting in the suppression of inflammatory gene transcription [24].

## 4. Food Components That Regulate Inflammation in Obese Adipose Tissue

**Figure 3:**
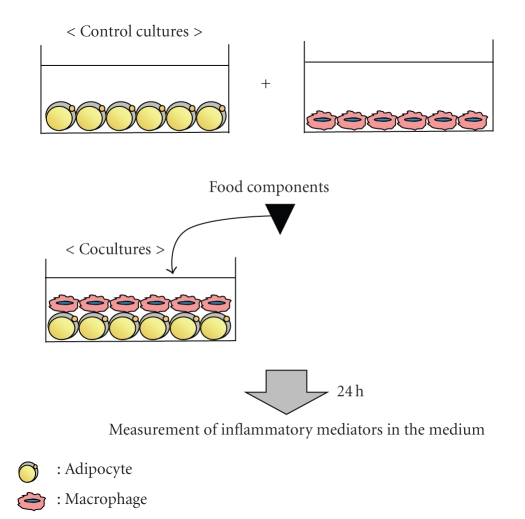
Coculture system of adipocytes and macrophages for the screening for anti-inflammatory food components.

On the basis of the strategy suggested above, our research group focused on the PPAR*γ*-dependent or PPAR*γ*-independent mechanisms to suppress the inflammatory mediators secreted by obese adipose tissues. For the screening of food components related to the former mechanism, our research group used the sensitive PPAR*γ* ligand assay system developed by modifying the luciferase reporter assay system [25] and has found several phytochemicals that act as PPAR*γ* agonists (Table 1). To evaluate the characteristics of food components that prevent obesity-induced inflammatory responses, we used the coculture system of adipocytes and macrophages, which is an* in vitro* model of obese adipose tissue infiltrated by macrophages (Figure 3).

### 4.1. PPAR*γ*-Dependent Action

Spices are derived from plants cultivated in temperate and tropical zones, and many of them have antioxidant, anticancer, antiobesity, and anti-inflammatory activities [26–28]. Several anti-inflammatory spice-derived components are reported to modulate inflammatory responses in adipose tissue and therefore improve obesity-related pathologies such as insulin resistance [29, 30]. 

Capsaicin, a spicy ingredient of hot peppers, has not only metabolic properties to induce thermogenesis and fat oxidation [26, 28] but also anti-inflammatory properties [31]. In the adipose tissue or adipocyte culture system, capsaicin inhibits the expression and secretion of IL-6 and MCP-1 from the adipose tissues and adipocytes of obese mice, whereas it enhances the expressions of the adiponectin gene and protein [29]. These actions of capsaicin are associated with NF-*κ*B inactivation, which is probably mediated by PPAR*γ* activation [29]. Moreover, capsaicin suppresses not only macrophage migration induced in an adipose-tissue-conditioned medium but also its activation to release proinflammatory mediators. It is also demonstrated that capsaicin administration *in vivo *improves obesity-induced insulin resistance [29].

Ginger, which is the rhizome of the plant *Zingiber officinale *Roscoe, is widely used as a spice and herbal medicine. 6-Shogaol is the main ginger-derived component, which has potent anti-inflammatory activities [32, 33]. Because 6-shogaol is a potent agonist of PPAR*γ*, it not only enhances the expressions of adiponectin and aP2 but also inhibits the TNF-*α*-induced downregulation of adiponectin expression in adipocytes [34].

Isoprenoids (terpenoids), which are present in many dietary and herbal plants [35], exhibit many biological effects: antitumor proliferation, anti-hypercholesteremia, and anti-diabetes [35–37]. Abietic acid (AA) and one of its derivatives, dehydroabietic acid (DAA), are diterpenes, which are both the major components of the rosin fraction of oleoresin synthesized by conifer species, such as grand fir (*Abies grandis*) and lodgepole pine (*Pinus contorta*) [38]. We have found that both AA and DAA have anti-inflammatory effects on macrophages, which are mediated by PPAR*γ* activation [25]. When DAA was administered with a high-fat diet to obese diabetic KK-Ay mice, DAA suppressed the production of proinflammatory mediators such as MCP-1 and TNF-*α*, increased that of adiponectin, and reduced the infiltration of macrophages into the adipose tissues of HFD-fed mice [39]. DAA can also strongly activate PPAR*α*, which is mainly involved in the control of lipid metabolism [40], and the fact that PPAR*α* agonists such as Wy-14643 can suppress inflammation in adipose tissues [41] suggests that DAA as a PPAR*α*/*γ* dual agonist is a valuable medicinal food-derived component for improving the inflammation caused by obesity and for controlling metabolic syndrome.

Auraptene (a monoterpene derivative), a citrus fruit compound contained mainly in the peel, is also a PPAR*α*/*γ* dual agonist [42, 43]. In adipocytes, auraptene regulates the transcription of PPAR*γ* target genes, induces the expression and secretion of adiponectin, and inhibits those of MCP-1 [42]. It is also observed that auraptene can suppress the inflammatory changes between adipocytes and macrophages and the macrophage infiltration into obese adipose tissues (Lin et al. unpublished data). Several reports have indicated that coapplication of PPAR*α* and PPAR*γ* agonists or treatment with dual agonists causes more efficient glucose uptake into adipocytes to decrease the blood glucose level without the increase in body weight [41, 44]. Further *in vivo* investigations are necessary to elucidate the inhibitory effect of auraptene on chronic systemic inflammation induced by obesity.

### 4.2. PPAR*γ*-Independent Action

Flavonoid is a general term for plant metabolites that have a C6-C3-C6 structure. Chalcone is the first product in the flavonoid biosynthesis pathway, which is catalyzed by chalcone isomerase, resulting in the flavanone naringenin. Most flavonoids are then metabolized to flavone, dihydroflavonol, flavonol, leucoanthocyanidin, catechin, and anthocyanidin by oxidation-reduction reaction. Over 4,000 flavonoids have been identified, many of which occur in vegetables and fruits. These flavonoids have been reported to have antiviral, antiallergic, antiplatelet, anti-inflammatory, antitumor, and antioxidant activities, and recently, they have attracted considerable interest because of their potential beneficial effects on obesity and metabolic syndromes.

Luteolin, a flavone that is present in medicinal plants and in some vegetables and spices, has been reported to exhibit antioxidant, anti-inflammatory, and antiallergy functions [45]. Recently, we have found that luteolin also inhibits low-grade chronic inflammation induced during the coculture of adipocytes and macrophages [17]. Luteolin does not affect I-*κ*B-*α* degradation and thus may not affect the NF-*κ*B activation. However, it inhibits the phosphorylation of JNK in the macrophages activated by the conditioned medium derived from adipocytes [17]. Because luteolin is not a PPAR*γ* agonist (Ando et al. unpublished data), luteolin may act on JNK directly or indirectly via a PPAR*γ*-independent mechanism.

Using the coculture system of adipocytes and macrophages, we have also found similar effects of naringenin chalcone, a type of flavonoid accumulated in tomato peels. Naringenin chalcone has only been reported as having antiallergic activities [46]; therefore, we examined its effect on the inflammatory changes associated with the interaction of adipocytes and macrophages. As in the case of luteolin, naringenin chalcone also suppresses the production of inflammatory mediators induced by the coculture of adipocytes and macrophages [12]. The flavanone naringenin, which is abundant in citrus fruits, also inhibits coculture-induced inflammation; however, the suppressive effect is more notable in naringenin chalcone [12]. However, unlike luteolin, naringenin chalcone and naringenin partly inhibit the degradation of I-*κ*B-*α* [12] and suppress the macrophage infiltration to hypertrophied adipocytes (Hirai et al. unpublished data). These three flavonoids do not serve as agonists of PPAR*γ* in the luciferase reporter assay (Hirai et al. unpublished data); thus, it is considered that they also affect the signaling molecules downstream of TLR4 directly or indirectly but independently of PPAR*γ* activation in macrophages.

Anthocyanins, another type of flavonoid found in red/purplish fruits and vegetables, including purple grapes, apples, blueberries, egg apples, and beans, are well-known antioxidants. These flavonoids have also been shown to have anti-inflammatory activity in obese adipose tissues, which is mediated by PPAR*γ*-independent mechanisms [47, 48]. Moreover, cyanidin 3-glucoside (C3G), a typical anthocyanin, downregulates the retinol binding protein 4, which is known to ameliorate insulin sensitivity in the white adipose tissue of diabetic KK-Ay mice [49]. Therefore, the C3G-induced improvement of insulin sensitivity may be associated with the inhibition of inflammatory mediators and stimulation of AMPK activity via PPAR*γ*-independent mechanisms [48].

Aside from flavonoids, a saponin aglycon, diosgenin, is also found to suppress the inflammatory mediators induced by the interaction of adipocytes and macrophages. Diosgenin is found in a variety of plants including fenugreek (*Trigonella foenum-graecum*) and roots of wild yam (*Dioscorea villosa*), and its extracts have been traditionally used to treat diabetes [50] and hypercholesterolemia [51]. Many researchers have shown that diosgenin has various biological functions, including anti-inflammation [52]. In our coculture system, diosgenin also inhibited the inflammatory changes via the downregulation of I-*κ*B-*α* degradation and JNK activation [53], which is independent of PPAR*γ* activation (Uemura et al. unpublished data).

6-Gingerol is another main ginger-derived component besides 6-shogaol. The structures of these two components are very similar and both are reported to inhibit TNF-*α*-mediated suppression of adiponectin in adipocytes; however, the mechanisms of their inhibitory effects are different; 6-gingerol inhibits JNK signaling pathways in TNF-*α*-induced adipocytes without affecting PPAR*γ* transactivation, whereas the anti-inflammatory action of 6-shogaol is PPAR*γ*-dependent [54]. These results suggest that slight structural differences may affect the affinity for PPAR*γ* and the inhibition of the JNK signaling pathways.

Although saturated fatty acids directly induce inflammatory responses in macrophages, long-chain *ω*-3 polyunsaturated fatty acids (PUFAs), such as docosahexaenoic acid (DHA) and eicosapentaenoic acid (EPA), are known as antiobesity and anti-inflammatory factors. Fish oil containing high concentrations of DHA and EPA is considered a good source of *ω*-3 PUFA. The prevention of high-fat or high-energy-diet-induced adipose tissue inflammation and remodeling by long-chain *ω*-3 PUFA is reported to be involved in PPAR*γ* activation [55, 56]. However, the anti-inflammatory mechanisms of PUFA action are diverse and involve PPAR*γ*-independent effects [57]. Furthermore, PUFA needs many cofactors such as folic acid, vitamins, tetrahydrobiopterin, minerals, and L-arginine for their physiological actions [58]. Hence, these cofactors should also be provided in adequate amounts to bring about the anti-inflammatory actions of *ω*-3 PUFA in obese adipose tissues.

## 5. Conclusions/Outlook

A growing number of studies strongly support that obesity-induced inflammation plays an important role in the development of obesity-related pathologies such as insulin resistance, cardiovascular diseases, type-2 diabetes, and some immune disorders. NF-*κ*B and JNK are important modulators of inflammatory gene expression downstream of TLR4 in obese adipose tissues, which are regulated by PPAR*γ*. All the food components described above are beneficial phytochemicals that ameliorate obesity-induced inflammatory responses and pathologies by suppressing the inflammatory signaling in a PPAR*γ*-dependent or PPAR*γ*-independent manner. In particular, PPAR*γ* agonists can directly reduce adipocyte size and induce the expression of anti-inflammatory cytokines, such as adiponectin [23]. Moreover, PPAR*γ* agonists have recently been reported to cause the polarization of adipose tissue macrophages to M2 phenotypes, resulting in the secretion of anti-inflammatory cytokines [59]. Thus, food components with PPAR*γ* agonistic activities may also contribute to the improvement of obesity-induced inflammation via adipose tissue remodeling associated with the phenotype switch of macrophages. Recently, it has been reported that a combination of bioactive compounds is very effective *in vivo* [60]. In particular, a combination of compounds exhibiting different mechanisms by which anti-inflammatory effects are exerted seems to be most efficient. Therefore, all the described phytochemicals in this review, which act as PPAR*γ* agonists, may be suitable for the treatment of metabolic syndrome together with other compounds that can suppress inflammatory responses in a PPAR*γ*-independent manner, by directly inhibiting NF-*κ*B or JNK signaling. As shown in Table 1, citrus fruits including oranges, grapefruits, lemons, and some limes, and fish oil from blue-skin fish such as sardine, herring, and albacore tuna, are the most available anti-inflammatory foods in the market. On the other hand, our daily intake of spices and herbs are still limited. Further studies on the effective amounts and forms of intake will help promote the development of all these functional foods in the world.

## Figures and Tables

**Table 1:** Phytochemicals that regulate obesity-induced inflammation.

Compound	Origin	PPAR*γ* dependency
Abietic acid	Pine rosin	dependent
Anthocyanin	Red/Purplish Fruit	independent
Auraptene	Citrus Fruit	dependent
Capsaicin	Hot pepper	dependent
Dehydroabietic acid	Pine rosin	dependent
Diosgenin	Fenugreek, Yam	independent
6-Gingerol	Ginger	independent
Isohumulone	Humulus lupulus hop	dependent
Isoprenoid	Herb	dependent
Luteolin	Herb, Spice	independent
Naringenin	Citrus Fruit	independent
Naringenin chalcone	Tomato peel	independent
PUFA	Fish oil	independent
Resveratrol	Red wine	dependent
6-Shogaol	Ginger	dependent
